# Cancer metabolism at a glance

**DOI:** 10.1242/jcs.181016

**Published:** 2016-09-15

**Authors:** Alexei Vazquez, Jurre J. Kamphorst, Elke K. Markert, Zachary T. Schug, Saverio Tardito, Eyal Gottlieb

**Affiliations:** 1 Cancer Metabolism Research Unit, Cancer Research UK Beatson Institute, Garscube Estate, Switchback Road, Glasgow G61 1BD, UK; 2 Institute of Cancer Sciences, University of Glasgow, Garscube Estate, Switchback Road, Glasgow G61 1QH, UK

**Keywords:** Cancer, Metabolism, Targeted therapy

## Abstract

A defining hallmark of cancer is uncontrolled cell proliferation. This is initiated once cells have accumulated alterations in signaling pathways that control metabolism and proliferation, wherein the metabolic alterations provide the energetic and anabolic demands of enhanced cell proliferation. How these metabolic requirements are satisfied depends, in part, on the tumor microenvironment, which determines the availability of nutrients and oxygen. In this Cell Science at a Glance paper and the accompanying poster, we summarize our current understanding of cancer metabolism, emphasizing pathways of nutrient utilization and metabolism that either appear or have been proven essential for cancer cells. We also review how this knowledge has contributed to the development of anticancer therapies that target cancer metabolism.

## Abstract (precis)

**Summary:** This article indicates metabolic pathways essential for cancer growth and survival, with an emphasis on anticancer therapies that are associated with such metabolic vulnerabilities.

## Introduction

Cancer cells typically proliferate from one aberrant cell to more than 10^9^ cells (the average number of cells in a tumor of ∼1 cm in diameter). To achieve and sustain that proliferative capacity, cancer cells must activate or enhance metabolic pathways (Lunt and Vander Heiden, 2011). These pathways use available nutrients to generate the metabolic precursors for cell anabolism, to satisfy the energy demand for cell maintenance and biosynthesis and to maintain the reduction–oxidation (redox) balance in the cell. The dry mass of mammalian cells is, for the most part, composed of proteins, lipids and nucleotides that are synthesized from metabolic precursors. Amino acids are the building blocks of proteins, acetyl-CoA is a precursor of lipids, and purines and pyrimidines form the backbone of nucleotides; these metabolic precursors are synthesized *de novo* from different nutrients.

**Figure:**
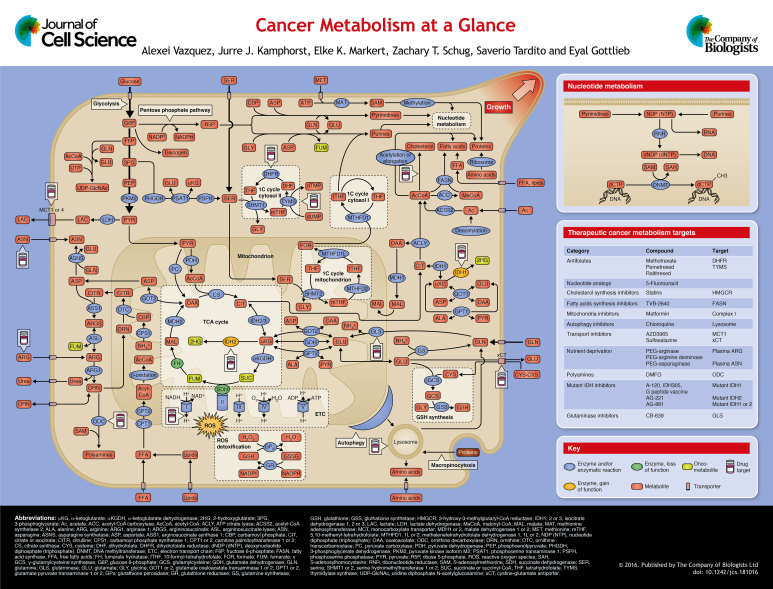

Here, we provide a concise graphical depiction of major metabolic pathways that are employed by cancer cells to sustain survival and proliferation. We also briefly describe these metabolic pathways from a nutrient-based perspective, and the outline follows the hierarchy of nutrient utilization by cancer cells. We start with glucose, the most abundant nutrient in the blood and, not surprisingly, the major contributor to the evolution of the metabolism of mammalian cells. We continue with glutamine, the second most consumed nutrient after glucose, followed by serine and other amino acids. We briefly discuss the role of fatty acid synthesis and highlight some recent discoveries about acetate as a potential source for the synthesis of lipids. Very concisely, we also go over the role of oxygen as both an electron acceptor supporting energy generation and the source of reactive oxygen species (ROS). Additionally, we review the role of scavenging during nutrient deprivation. Finally, in a departure from our nutrient-based perspective, we briefly discuss the concept of oncometabolites that link core metabolic pathways with cancer signaling and epigenetics.

## Glucose

Normally, most of the glucose consumed by cells is catabolized through glycolysis to pyruvate, which is transported to the mitochondria. In the mitochondria of aerobic cells, pyruvate fuels the tricarboxylic acid (TCA) cycle and the electron transport chain (ETC), where oxidative phosphorylation takes place. Glucose catabolism coupled to oxidative phosphorylation has a high energy yield in the form of ATP. Cancer cells, paradoxically, convert much of the pyruvate into lactate, which is then excreted to the extracellular medium (see poster). The catabolism of glucose into lactate has an extremely low energy yield and, consequently, cancer cells require a high glucose consumption rate to satisfy their energy and anabolic demands. A high rate of glucose catabolism into lactate (glucose fermentation) is the most ubiquitous metabolic phenotype seen across cancer cells and was first reported by Warburg (1956). The transport of lactate out of cells is facilitated by monocarboxylate transporters (MCTs) and, given that they are required to sustain the high rates of glycolysis in cancer cells, MCTs are candidate targets for cancer therapy (Doherty and Cleveland, 2013) (see poster table).

In cells, glucose is also a major carbon source for biosynthesis. The pyruvate derived from glucose contributes to the synthesis of acetyl-CoA, a precursor of fatty acid, lipid and cholesterol synthesis. Pyruvate also participates in the synthesis of the non-essential amino acids aspartate and asparagine, via the activities of pyruvate carboxylase and glutamate and oxaloacetate transaminases (GOT1 and GOT2). Other intermediate metabolites of glycolysis are also biosynthetic precursors. Glucose 6-phosphate (G6P) is the branching point from glycolysis to the oxidative branch of the pentose phosphate pathway (PPP), which generates the ribose group required for the synthesis of nucleotides. The PPP is also a major pathway for NADPH generation. Finally, 3-phosphoglycerate (3PG) is the branching point for the synthesis of the non-essential amino acid serine (discussed below).

In summary, cancer cells use the catabolism of glucose through glycolysis as a major energy-generating pathway. In addition, several biosynthetic molecules and NADPH are generated from glucose.

## Glutamine

Glutamine, alongside alanine, redistributes nitrogen and carbons between source- and sink-organs for these elements. In accordance with its pleiotropic functions, glutamine is the most abundant circulating amino acid in humans. Under *in vitro* cell culture conditions, glutamine is the second most consumed nutrient after glucose, and its consumption exceeds its demand for protein synthesis (Jain et al., 2012). However, a high proportion of the consumed glutamine is generally de-amidated and released into the medium as glutamate (Bannai and Ishii, 1988; Jain et al., 2012; Timmerman et al., 2013). The export of glutamate is often coupled to cystine import through the xCT antiporter (Bannai and Ishii, 1988; Timmerman et al., 2013). Glutamate is produced from glutamine by several amidotransferase-catalyzed reactions, while the amidic nitrogen of glutamine is transferred to metabolic intermediates, such as asparagine, nucleotides and glucosamine phosphate. Additionally, glutaminases (GLS and GLS2) hydrolyze glutamine to glutamate and inorganic ammonia. Inhibition of GLS has shown anti-tumor effects in several cancer models, and one such inhibitor is currently under clinical investigations (Gross et al., 2014; Jacque et al., 2015; Shroff et al., 2015). However, the efficacy of GLS-targeted therapies appears to be affected by cell culture conditions (Davidson et al., 2016). In addition, xCT, which exports glutamate from the cell, had a pro-tumorigenic effect in glioma patients and triple negative breast cancer models (Robert et al., 2015; Timmerman et al., 2013).

Glutamine-derived glutamate can also be produced in excess of its demand for protein synthesis. The excess glutamate is either exported to the extracellular medium (Jain et al., 2012) or converted into α-ketoglutarate (αKG) by glutamate dehydrogenase (GDH) or transaminases. αKG can be metabolized in the TCA cycle, either oxidatively or reductively, and, hence, it contributes to anaplerosis, the replenishing of TCA cycle metabolites. The synthesis of citrate from αKG through reductive carboxylation has been reported to be essential for growth of cancer cells with mitochondrial defects (Mullen et al., 2012), suggesting that these cancers could be distinctively sensitive to the inhibition of glutamine metabolism.

Glutamine synthetase, which catalyzes the ATP-dependent condensation of glutamate and ammonia, is the only enzyme able to synthesize glutamine. In hepatocellular carcinoma (HCC), activating mutations in β-catenin increase the expression of the glutamate transporter (EAAT2, also known as SLC1A2), as well as of glutamine synthetase (Cadoret et al., 2002; Dal Bello et al., 2010). Glutamine synthetase inhibition and glutamine depletion hamper the growth of HCC xenografts (Chiu et al., 2014), suggesting that high glutamine levels could be instrumental for the metabolism of liver tumors, independently of its anaplerotic function. In line with tissue-specific differences in glutamine utilization (Yuneva et al., 2012), the availability of circulating glutamine is limited. Accordingly, glutamine synthetase activity in either stem-like cancer cells or in neighboring astrocytes is required for glutamine supplementation to glutamine-synthetase-negative glioblastoma cells (Tardito et al., 2015). The anaplerotic contribution of glutamine has been shown to sustain the anabolic metabolism of cultured cancer cells derived from various tissues. On these bases, GLS has been identified as a potential therapeutic target for ‘glutamine-addicted’ tumors. However, the validity of this phenotype awaits confirmation in pre-clinical models where the glutaminase-dependent anabolic contribution appears to be downsized (Davidson et al., 2016; Tardito et al., 2015).

Thus, the pro-growth effect of glutamine cannot be solely explained by carbon metabolism, and the central role of this pleiotropic amino acid in nitrogen metabolism, as well its contribution to signaling events, must be considered to fully understand the multifaceted relationship between glutamine and cancer.

## Serine and one-carbon metabolism

The non-essential amino acid serine can be either imported from the medium or synthesized from the glycolytic intermediate 3PG. The first step of serine synthesis is catalyzed by 3PG dehydrogenase (PHGDH), an enzyme that is often genetically amplified in breast cancers (Possemato et al., 2011) and melanoma (Locasale et al., 2011). This has motivated the investigation of PHGDH as a target for cancer therapy. Serine is the third most consumed metabolite by cancer cells after glucose and glutamine (Dolfi et al., 2013; Jain et al., 2012). A significant amount of serine is converted into glycine (Maddocks et al., 2013; Tedeschi et al., 2015a) through the activity of cytosolic or mitochondrial serine hydromethyltransferases (SHMT1 and SHMT2). In this reaction, serine releases a one-carbon unit to the one-carbon pool. In principle, glycine could also contribute to the one-carbon pool through the glycine cleavage system. However, the available data indicates that serine is the major donor of one-carbon units in cancer cells and that glycine cannot substitute for serine (Labuschagne et al., 2014).

The one-carbon unit donated by serine is utilized in the biosynthesis of thymidine monophosphate (dTMP) and purines, and the available evidence suggests that specific one-carbon pools are located in different cellular compartments (see poster). For instance, the one-carbon stock for dTMP synthesis is generated in the cytosol in a cyclical pathway involving SHMT1, thymidylate synthase (TYMS) and the co-enzyme folate in the form of dihydrofolate (DHF) and tetrahydrofolate (THF) (see poster, 1C cycle cytosol II; Oppenheim et al., 2001). Indeed, TYMS and DHF reductase (DHFR) are the targets of some of the earliest anti-cancer drugs, such as 5-fluoracil and methotrexate (Wilson et al., 2014) (see poster table).

In contrast, the one-carbons required for purine synthesis are generated in the mitochondria (see poster, 1C cycle mitochondrion, Lewis et al., 2014). The mitochondrial one-carbon units are transferred to and from THF via SHMT2, 5,10-methenyl-THF dehydrogenase 2 (MTHFD2) and 10-formyl-THF synthase (MTHFD1L), releasing the one-carbon unit as formate. Formate is then transported to the cytosol where it is ligated to THF by cytosolic 10-formyl-THF synthase (MTHFD1) to generate the cytosolic 10-formyl-THF that transfers the one-carbon units to purines (see poster, 1C cycle cytosol I). MTHFD2 is expressed mostly in embryonic and cancer tissues, whereas in the mitochondria of normal adult tissues, MTHFD2 activity is carried out by the product of a different gene, MTHFD2L (Bolusani et al., 2011). This observation points to MTHFD2 as a potential target for cancer therapy (Nilsson et al., 2014; Tedeschi et al., 2015b). SHMT2 has been shown to drive cancer cell growth in different contexts (Kim et al., 2015; Ye et al., 2014). Furthermore, high SHMT2 in cancer cells creates a dependency on glycine clearance that could be exploited for anticancer therapy (Kim et al., 2015).

Taken together, the discovery of the genes coding for mitochondrial one-carbon metabolism enzymes has led to a significant advance in our understanding of mitochondrial one-carbon metabolism in normal and cancer cells. It remains to be elucidated whether new drugs specifically targeting mitochondrial one-carbon metabolism have anticancer activity.

## Methionine and methylation

In addition to nucleotide metabolism, the one-carbon pool is required for methylation processes, which are crucial for epigenetic regulation of gene expression. The essential amino acid methionine can be either imported into cells or recycled through the methylation cycle. In the canonical methylation cycle, methionine is converted into S-adenosyl-methionine (SAM) through the activity of methionine adenosyltransferase transferase (MAT). Here, SAM provides a methyl group and is converted into S-adenosylhomocysteine (SAH). SAH is then hydrolyzed to adenosine and homocysteine. Finally, methionine synthase transfers a one-carbon unit from 5-methyl-THF to homocysteine, thereby regenerating methionine.

*In vitro* data from different cell lines indicate that this methylation cycle is truncated in cancer cells and that methionine synthase activity is negligible compared with the rate of methionine consumption (Mentch et al., 2015; Shlomi et al., 2014; Tedeschi et al., 2015a). Homocysteine is not recycled back to methionine, but is instead secreted from cells (Shlomi et al., 2014). However, this *in vitro* evidence does not exclude the possibility that, *in vivo*, methionine synthase provides a higher contribution to the methylation cycle, particularly during conditions of nutrient starvation.

## Arginine, ornithine and the urea cycle

Both the non-essential amino acid arginine and the non-proteogenic amino acid ornithine contribute to cancer cells beyond protein synthesis. Arginine and ornithine can be imported into cells, and arginine can be converted into ornithine through the activity of arginase (ARG1 and ARG2). Ornithine, a precursor of polyamine synthesis, is essential for cell proliferation (Pegg, 2009). The first step of polyamine synthesis is catalyzed by ornithine decarboxylase (ODC1). 2-Difluoromethylornithine is an irreversible inhibitor of ODC1; however, it failed as a single agent in multiple clinical trials (Casero and Marton, 2007). Arginine and ornithine are both intermediate metabolites of the urea cycle, although it is not clear whether this cycle plays a significant role in cancer cell metabolism. In cancer cells with fumarate hydratase deficiency, the urea cycle step catalyzed by argininosuccinate lyase (ASL) manifests a high rate of synthesis of argininosuccinate from fumarate and arginine, allowing these cells to utilize, and decrease the levels of, the fumarate generated by the truncated TCA cycle, at the expense of an increased demand for arginine (Adam et al., 2013; Zheng et al., 2013).

In many types of cancers, the expression of argininosuccinate synthase (ASS1) is silenced epigenetically, rendering the cells dependent on an exogenous supply of arginine (Qiu et al., 2015). ASS1 converts aspartate and citrulline into argininosuccinate, and the loss of ASS1 supports proliferation by increasing the availability of aspartate for nucleotide biosynthesis (Rabinovich et al., 2015).

## The sources of acetyl-CoA and fatty acid synthesis

Acetyl-CoA is the precursor for the synthesis of fatty acids and cholesterol. Both glucose and glutamine can contribute to the generation of acetyl-CoA. Acetate has recently been shown to be yet another source of acetyl-CoA for many different cancer types, including breast, prostate, liver, primary glioblastomas and brain metastases. Some of these cancerous tissues incorporate acetate into fatty acids to support biomass production, whereas others have been shown to use acetate to fuel the TCA cycle (Comerford et al., 2014; Kamphorst et al., 2014; Mashimo et al., 2014; Schug et al., 2015). Mechanistically, acetate is ligated to CoA by the acyl-CoA synthetase short-chain family member 2 (ACSS2), an enzyme that is upregulated during conditions of metabolic stress such as low lipid availability and hypoxia. As such, acetate might become a crucial nutritional source in poorly vascularized regions of tumors.

The acetyl-CoA generated from glucose, glutamine or acetate (or potentially other nutritional sources) supplies the cholesterol and fatty acids synthesis demand of cancer cell growth. Cholesterol synthesis from acetyl-CoA proceeds through the mevalonate pathway. Statins are inhibitors of 3-hydroxy-3-methylglutaryl-CoA reductase (HMGCR), the limiting step of the mevalonate pathway, and are used to lower cholesterol levels in patients with cardiovascular diseases. The epidemiological observation that long-term treatment with statins is associated with a reduced incidence of cancer stimulated research on the use of statins for cancer prevention (Baandrup et al., 2015; Boudreau et al., 2010). However to date, clinical trials designed to specifically test cancer prevention by statin treatment have not been conclusive (Bertagnolli et al., 2010; Cardwell et al., 2015).

The tumor-promoting effects of enhanced fatty acid synthesis were first appreciated in the 1990s when fatty acid synthase (FASN) expression was identified as prognostic marker of aggressive breast cancers (Kuhajda et al., 1994). Since then, many FASN inhibitors have been developed, but their potential on-target toxicity remains a concern in clinical trials (Pandey et al., 2012), warranting investigation into other anti-lipid metabolism drugs. Another promising target in the field is stearoyl-CoA desaturase (SCD) (Mason et al., 2012), for which multiple studies have identified a crucial role in tumor growth (Fritz et al., 2010; Scaglia and Igal, 2008). Biochemically, given that SCD inhibition is often rescued by oleic acid supplementation (Griffiths et al., 2013), SCD maintains a balance between the saturated and unsaturated fatty acid content within the phospholipid pools. Loss of this balance often leads to ER stress and apoptosis activation (Potze et al., 2016; Young et al., 2013). Besides lipid synthesis, lipid breakdown also appears to be a common feature of cancer development. The tendency of ovarian cancers to metastasize to the omentum has been shown to be driven by crosstalk between adipocytes and ovarian cancer cells (Nieman et al., 2011). Cytokine signaling by the ovarian cancer cells induces adipocytes to mobilize lipid stores. The released fatty acids are scavenged by the invading ovarian cells and, through the expression of fatty acid binding protein 4 (FABP4), fatty acids are channeled into the fatty acid oxidation machinery for energy production. The uptake of fatty acids not only by the tumor cells but also the stromal compartment can affect tumorigenesis. Many stromal and fat cells of human breast tumors strongly downregulate the fatty acid scavenging protein CD36 (DeFilippis et al., 2012). Loss of CD36 in the stromal compartment of invasive breast cancers induces an extracellular matrix deposition and increase in mammographic density, which is associated with poorer prognosis. Conversely, lipoprotein lipase (LPL) and CD36 are frequently highly expressed in breast cancer tissue, and their overexpression in breast cancer cell lines activates a lipolytic pathway that enhances growth in culture (Kuemmerle et al., 2011). In more aggressive tumors, monacylglycerol lipase (MGLL)-dependent hydrolysis of monoacylglycerols generates fatty acids used to produce signaling lipids, such as lysophosphatidic acid and prostaglandins, that promote migration, cancer cell survival and tumor growth (Nomura et al., 2010). Furthermore, the specific activation of adipose triglyceride lipase (PNPLA2; also known as ATGL) in the white adipose tissue and skeletal muscle of cancer patients has been linked to cachexia, suggesting that inhibition of lipases might help alleviate the devastating problems associated with it (Das et al., 2011).

Taken together, the targeting of lipid metabolism pathways holds great promise and as new targets emerge, it will be interesting to see how they synergise with current therapies.

## Metabolic scavenging during nutrient starvation

Tumor areas can experience episodes of limited nutrient supply due to poor perfusion to the degree that nutrient availability is insufficient for maintaining proliferation or even survival. Additionally, some tumors are chronically poorly perfused due to low vascularization and high interstitial pressure; a good example of this is pancreatic ductal adenocarcinoma (PDAC) (Koong et al., 2000; Neesse et al., 2011). A well-described alternative mode of nutrient acquisition during these periods of starvation is autophagy or ‘self-cannibalism’ (Rabinowitz and White, 2010). An important function of autophagy in normal cells is the removal of damaged and dysfunctional organelles. It is a catabolic pathway used by cells to degrade cytoplasmic content and organelles, and recycle their components. The process utilizes autophagosomes that eventually fuse with lysosomes for content degradation and the subsequent release of the breakdown products. During nutrient starvation, the breakdown products generated by autophagy (amino acids, fatty acids, sugars, and nucleosides) help sustain energy production and synthesis of essential cellular building blocks. In tumor cells, autophagy has been found to be particularly important for maintaining survival during nutrient stress (Guo et al., 2011; Yang et al., 2011).

Although it can support cell survival during episodes of starvation, autophagy is inherently incapable of facilitating tumor growth, as it only recycles or consumes the intracellular biomass. To support proliferation, exogenous substrates are required. Recently, it was found that cancer cells, particularly those with a Ras mutation, can internalize extracellular proteins through macropinocytosis (Commisso et al., 2013). This is an endocytic process by which extracellular fluid and its components are engulfed by the plasma membrane, leading to the budding of macropinosomes in the cytoplasm (see poster). Like autophagosomes, macropinosomes also fuse with lysosomes and the degraded content is released to support metabolism. In the study by Commisso et al., cell consumption of albumin was found to reduce the dependence on free glutamine (Commisso et al., 2013), and a later study confirmed that macropinocytosis occurs in human tumors (Kamphorst et al., 2015). Additionally, this process enables pancreatic cancer cells to proliferate in medium lacking essential free amino acids (Kamphorst et al., 2015). Although the exact signaling events downstream of Ras that are responsible for induction of macropinocytosis remain elusive, recently, remarkable connections were found to the nutrient sensor mammalian target of rapamycin complex 1 (mTORC1) and its regulation of lysosomal processing. One study showed that, whereas under nutrient-replete conditions mTORC1 inhibition suppressed growth, under limited amino acid availability mTORC1 inhibition actually enhances lysosomal degradation of endocytosed proteins and hence promotes proliferation (Palm et al., 2015). Another study found that, regardless of mTORC1 activity, PDAC cells have heightened lysosome biogenesis compared to their non-tumor counterparts (Perera et al., 2015).

In summary, some cancer cells survive and even grow during primary nutrient starvation by maintaining metabolic activity through catabolizing both intracellular and extracellular macromolecules. This metabolic ‘scavenging’ appears important for the growth of a subset of tumors, such as PDAC. Conceivably, it can also form a general mechanism of resistance to antiangiogenic therapies or other nutrient deprivation-based strategies. As all macromolecule catabolism pathways converge at the lysosome, inhibiting the functioning of this organelle could provide a therapeutic opportunity, either as a mono- or combination-therapy. Recently, encouraging results were obtained in a phase I and II trial, in which hydroxychloroquine (which prevents acidification of lysosomes) in combination with gemcitabine led to a significant increase in mean overall survival in PDAC (Boone et al., 2015).

## Oxygen and reactive oxygen species

From the early observations by Otto Warburg it is known that although cancer cells exhibit high glycolysis rates, they still retain some mitochondrial oxidative phosphorylation activity (Koppenol et al., 2011). Today it is well established that cancer cells satisfy part of their energy demand from the oxidation of glucose, glutamine and other nutrients coupled to the electron transport chain (ETC) (Fan et al., 2013; Hensley et al., 2016), using oxygen as the final electron acceptor (see ETC pathway on the poster). The ETC complex I inhibitor metformin is currently under investigation for cancer treatment (Pollak, 2012).

Tumor cells also periodically experience hypoxic conditions (i.e. low oxygen availability). Hypoxia results in increased glycolysis and, correspondingly, decreased oxidative phosphorylation that is caused either directly, due to the limitation of oxygen, or indirectly, by the activation of the hypoxia inducible transcription factor 1 α (HIF1A) (Kim et al., 2006; Papandreou et al., 2006). Moreover, various oxygen-dependent anabolic reactions might be affected by the lack of oxygen, including the lipogenic enzyme SCD, which introduces a double bond into the 18-carbon fatty acid stearic acid, leading to the production of the mono-unsaturated fatty acid oleic acid, one of the most abundant fatty acids in cells (Kamphorst et al., 2013; Young et al., 2013). This desaturation reaction requires molecular oxygen, and so is significantly decreased in hypoxia. Consequently, cells become more dependent on scavenging oleic acid from exogenous sources to maintain a proper desaturation index (Kamphorst et al., 2013).

Mitochondrial oxygen metabolism is linked to the generation of reactive oxygen species (ROS) (Sabharwal and Schumacker, 2014), which, at high levels, can damage nucleotides, proteins and lipids, so impairing cell viability. To counteract this, mammalian cells have different pathways for ROS detoxification. In cancer cells, glutathione (GSH) oxidation–reduction coupled to NADPH reduction–oxidation is a major pathway for ROS detoxification (see ROS detoxification on the poster). GSH is synthesized from the amino acids cysteine, glutamate and glycine (see poster). GSH oxidation by GSH peroxidase is coupled to the turnover of hydrogen peroxide (H_2_O_2_), a major ROS byproduct of mitochondrial oxidative phosphorylation. Oxidized glutathione (GSSG) is then reduced back to GSH by GSH reductase coupled to NADPH oxidation. ROS turnover through this pathway requires a supply of NADPH. As discussed above, NADPH can be generated from glucose from the pentose phosphate pathway or from serine via one-carbon metabolism.

## Oncometabolites

Beyond elucidating some of the catabolic, energetic and anabolic requirements for cancer growth and survival, studies over the past decade have introduced a new link between metabolic abnormalities and cancer progression. Generally termed ‘metabolic signaling’ (Gottlieb and Tomlinson, 2005), it was initiated by the identification of an intracellular signal transduction cascade that is mediated by TCA cycle metabolites (Isaacs et al., 2005; Selak et al., 2005). In tumors that have lost the mitochondrial tumor suppressor enzymes succinate dehydrogenase or fumarate hydratase, the respective accumulation of succinate or fumarate has been shown to inhibit the enzymatic activity of αKG-dependent dioxygenases, which hydroxylate HIF1A and target it for degradation. Hence, in tumors that have lost succinate dehydrogenase or fumarate hydratase, HIF1A is activated under normoxic conditions, resulting in pseudohypoxia (Frezza et al., 2011). More recently, it has been shown that succinate and fumarate are also potent modifiers of the epigenome through their role in inhibiting other family members of the αKG-dependent dioxygenases, namely histone or DNA demethylases (Nowicki and Gottlieb, 2015). With the discovery of isocitrate dehydrogenase (IDH1 and IDH2) mutations in cancer and the associated accumulation of an unique *de novo* product, 2-hydroxyglutarate (2HG), the phrase ‘oncometabolites’ was coined (Dang et al., 2009). 2HG has since been shown to be a potent epigenome regulator and this is currently considered to be its major pro-oncogenic role (Nowicki and Gottlieb, 2015).

In summary, the discovery of IDH1 or IDH2 mutations in human cancers has opened a window of opportunity for targeted therapies against cancers harboring these mutant enzymes. The hypothesis is that if 2HG is an oncometabolite, the inhibition of its production should inhibit tumor growth. Ongoing clinical trials are testing the anticancer activity of specific inhibitors of IDH1 and IDH2 mutant enzymes and a positive outcome would support the concept of oncometabolites, thereby providing a productive new example of targeted therapy against cancer metabolism.

## Conclusions

Cancer metabolism has been the target of cancer therapy since the early days of chemotherapy, with antifolates, for example, among the first targeted treatments. Our understanding of cancer metabolism has advanced significantly in recent years and is being used for the development of novel targeted therapies (Olivares et al., 2015). There are, however, several outstanding questions in the field of cancer metabolism that are and will be the subject of further research (see Box 1).

Box 1. Burning questions in cancer metabolism.

- What is the functional relevance and selective advantage of mitochondrial one-carbon metabolism?
- What is the role of acetate in the interaction between metabolism and epigenetics in cancer?
- Under which genetic and environmental conditions does the requirement of tumors for glutamine-derived carbon outweigh their demand for glutamine-derived nitrogen?
- How is macropinocytosis regulated and how important is it in supporting growth *in vivo*?
- What are the downstream factors mediating the tumorigenic activity of oncometabolites?
